# PEE POWER^®^ urinal II – Urinal scale-up with microbial fuel cell scale-down for improved lighting

**DOI:** 10.1016/j.jpowsour.2018.02.047

**Published:** 2018-07-15

**Authors:** Xavier Alexis Walter, Irene Merino-Jiménez, John Greenman, Ioannis Ieropoulos

**Affiliations:** Bristol BioEnergy Centre (B-BiC), Bristol Robotics Laboratory, T-Block, Frenchay Campus, University of the West of England (UWE), Bristol, BS16 1QY, United Kingdom

**Keywords:** Off-grid sanitation, Scale-up, Urine, Sustainable energy, Microbial fuel cells, PEE POWER^®^

## Abstract

A novel design of microbial fuel cells (MFC) fuelled with undiluted urine was demonstrated to be an efficient power source for decentralised areas, but had only been tested under controlled laboratory conditions. Hence, a field-trial was carried out to assess its feasibility for practical implementation: a bespoke stack of 12 MFC modules was implemented as a self-sufficient lit urinal system at UK's largest music festival. Laboratory investigation showed that with a hydraulic retention time (HRT) of 44 h, a cascade of 4 modules (19.2 L displacement volume) was continuously producing ≈150 mW. At the same HRT, the chemical oxygen demand (COD) was reduced from 5586 mg COD·L^−1^ to 625 mg COD·L^−1^. Field results of the system under uncontrolled usage indicate an optimal retention time for power production between 2h30 and ≈9 h. When measured (HRT of ≈11h40), the COD decreased by 48% and the total nitrogen content by 13%. Compared to the previous PEE POWER^®^ field-trial (2015), the present system achieved a 37% higher COD removal with half the HRT. The 2016 set-up produced ≈30% more energy in a third of the total volumetric footprint (max 600 mW). This performance corresponds to ≈7-fold technological improvement.

## Highlights

• First field trial of a novel design of scalable membraneless MFCs.
• The MFC stack did not require any energy input to function.
• 30% more power from 1/3 of the total volumetric footprint compared to 2015.
• 92% higher COD removal from half the retention time compared to 2015.
• 88% Chemical Oxygen Demand removal at 44 h HRT.

## Graphical abstract

**Figure:**
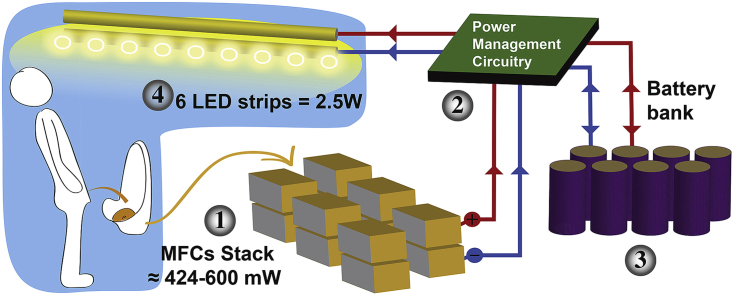

## 1 Introduction

Microbial fuel cells (MFCs) are energy transducers, first reported in 1911 [1], which produce electricity through the bio-electro-oxidation of organic compounds. Over the last two decades, during which research in the field has intensified, oxygen has become the most common end-terminal electron acceptor in the cathode, due to its availability and high redox value [[2], [3], [4], [5]]. Individual MFCs produce relatively low levels of power, hence a plurality of units must be assembled in stacks to reach useful power levels [6,7]. However, deploying MFC stacks in real environments presents two main challenges: cost and complexity. Self-stratifying membraneless MFCs (SSM-MFC) have been shown to address such a dual need [8]. At the same time, SSM-MFCs have demonstrated the capacity to be scaled-up in size, up to a certain extent, without significant power density losses [8]. The principle behind SSM-MFCs is to employ the ability of microorganisms to vertically self-stratify across physicochemical conditions of any given water column (e.g. lake, or urine). In such a column, cathodes are placed on the top whilst the anodes are placed on the bottom layers. This configuration is somewhat similar to single compartment MFCs with multiple cathodes and anodes. The main difference being that the cathode is partially submerged into the electrolyte and occupies about half of the available urine column's depth. Since the upper layer of the urine column (i.e. the catholyte) is separated from the bottom layer (i.e. the anolyte) by a bioelectrochemical gradient, an interpretation could be that this gradient is a transient membrane renewed after each feeding pulse. This aspect led to the naming of this type of MFC a SSM-MFC: Self-stratifying membraneless MFCs. However, until now this type of MFC has only been tested under controlled laboratory conditions [8,9].

This study was carried out from an implementation perspective and focused on the generation of energy from urine. Employing urine as fuel presented several advantages, one of which is that MFCs can be fuelled directly with neat urine (i.e. without any dilution nor pre-treatment) [10], a waste stream representing 75% of the nitrogen found in domestic wastewater [11]. Such a technology could lower the burden on wastewater treatment plant. By integrating the MFC technology in waterless urinals, the energy consumption of wastewater treatment plants would be reduced, but with useful energy being produced near the source (e.g. for charging smart phones, providing light or automation of sanitary peripherals) [9,12,13].

Although numerous reports focus on improving the technology, there is a relatively small number of field trial studies describing pilot-scale MFC systems deployed under real conditions. The first practical demonstration, which can be considered as field-trial, is to be found in the world of electronic/robotic with the “gastrobot” [14], or the Ecobot series [6,15]. Along these prototypes demonstrating the potential of MFC to act as power source, another series of early successful trials demonstrated the use of benthic and marine MFCs to power sensors [[16], [17], [18]]. As discussed in these previous studies, the implementation of MFC implies the use of power management circuitry (e.g. DC-DC converters, power harvester, capacitors/batteries) to match the MFC's lower but constant energy production to the application's higher, and sometimes intermittent, energy needs. Aside robotics and marine environments, wastewater treatment is the other main niche in which research focuses. A recent review documents the performance of litre-scale MFCs treating real wastewater at continuous flow mode, thus illustrates the potential for implementation and technology readiness [19]. Up until 2010, only three pilot-scale trials had been tested [20]. The first large pilot-scale MFC stack treating wastewater was fuelled by brewery waste in Yatala (Queensland, Australia), where twelve 3 m high MFC modules with a total volume of 1 m^3^ were used [20]. Since then, several studies have been conducted “out-of-the-lab” and/or at a pilot-scale: MEC for hydrogen production from wastewater [21], benthic microbial fuel cells [18], MFC in constructed wetland for wastewater treatment [22,23] and prototypes to be integrated in wastewater treatment plants [19,[24], [25], [26], [27], [28], [29], [30], [31]], Floating MFCs combined with plants that act as autonomous sensors able to transmit a signal in natural water bodies [32], and MFC-based urinal system [13], have also been reported.

The aim of the present trial was to assess the feasibility of SSM-MFC to be deployed as an electricity-generating sanitation solution in decentralised areas for periodic usage [9]. In order to test SSM-MFC in real conditions, the site for the trial should (i) have a need for lighting, automation or device charging, (ii) have a high number of users to provide the fuel and (iii) have a need for waterless *in-situ* treatment. Due to the existing collaboration between the Bristol BioEnergy Centre and the Glastonbury Music Festival, the PEE POWER^®^ urinal was tested in real conditions for a short period of time (3 weeks in total, including the 6 days of the music festival) at Glastonbury 2016. The Glastonbury Music Festival attracts approximately 250,000 people (festival goers and staff) and has a strong environmental agenda, with a high interest for on-site treatment and off-grid power. However, for such a purpose the system had to be re-designed to meet the on-site needs (i.e. a very high number of users per day; automatic feeding) [13]. Practically this meant scaling-up the whole system, whilst keeping the MFC modules smaller than the ones used in the PEE POWER^®^ urinal of 2015, adapting a passive feeding mechanism, and setting-up the appropriate energy management system, to harvest the energy and power the higher-energy consuming lights. Compared to its predecessor [13], the aim was to provide twice the amount of lighting (the urinal was twice the size) with a smaller footprint MFC system (<1/3 vol by comparison). Overall, the present study provides (i) results from laboratory investigation, (ii) performance under real conditions-of-use at the festival (iii) a self-sufficient system comprising MFCs and peripherals delivering a service to the users.

## 2 Materials and methods

Prior the trial, larger MFC modules were initially tested under laboratory conditions and then the system was assembled and tested on site, at the Glastonbury Music Festival 2016.

### 2.1 MFC modules construction and cascades configurations

#### 2.1.1 Scaling-up the module size

The SSM-MFC modules employed had a similar design as the ones previously described [8,9]. The cathodes were in the aerobic upper layers of the urine column whilst the anodes were in the anaerobic lower layer of the column. Due to the amount of fuel to be treated being greater (up to 1000 L d^−1^), the size of individual modules was increased by a factor of 2 in length and width and the modules had the following external dimensions: 400 mm length, 300 mm width and 170 mm height (“large module”). A total of 38 MFCs were inserted within this volume and all were electrically connected in parallel. The total footprint volume of a module was 20.4L, of which 11.2L was occupied by the MFCs (i.e. internal volume). The rest of the volume was occupied by air since the upper 5 cm of each box served as a support for upstream modules, resulting in a displacement volume of 4.8L of electrolyte. The modules were inoculated with the output waste-stream from other urine-fuelled MFCs. The first module tested had a feeding regime of 1.25L neat urine pulses every 2 h. Urine (pH between 8.5 and 9.2) was collected and pooled daily, from anonymous individuals with no known previous medical conditions.

#### 2.1.2 Laboratory cascade configuration

Two cascades of 4 modules each were assembled and initially tested in the laboratory. A cascade is defined as a set of modules where the output of one is feeding into the input of the next one. Hence a cascade is a series of modules treating the same fuel. Both cascades were fed from the same gravity-feed mechanism which was pulsing 3.4L every 2 h through two outputs, one for each cascade. As such, each cascade was receiving 1.7L every 2 h, unless otherwise stated. As the pulse-feed regime and the power produced are directly related [8], when the feeding rate was increased, so was the load. With this same hydraulic configuration, three electrical connections were tested: (i) both cascades were electrically independent and all four modules within each cascade connected in parallel, (ii) all four modules within each cascade were connected in parallel, and the two cascades connected in series, and (iii) the modules were connected in parallel by pairs, and the four pairs were then connected in series. The applied loads were consequently adapted to the electrical configurations.

#### 2.1.3 Glastonbury 2016 system configuration

**Fig. 1:**
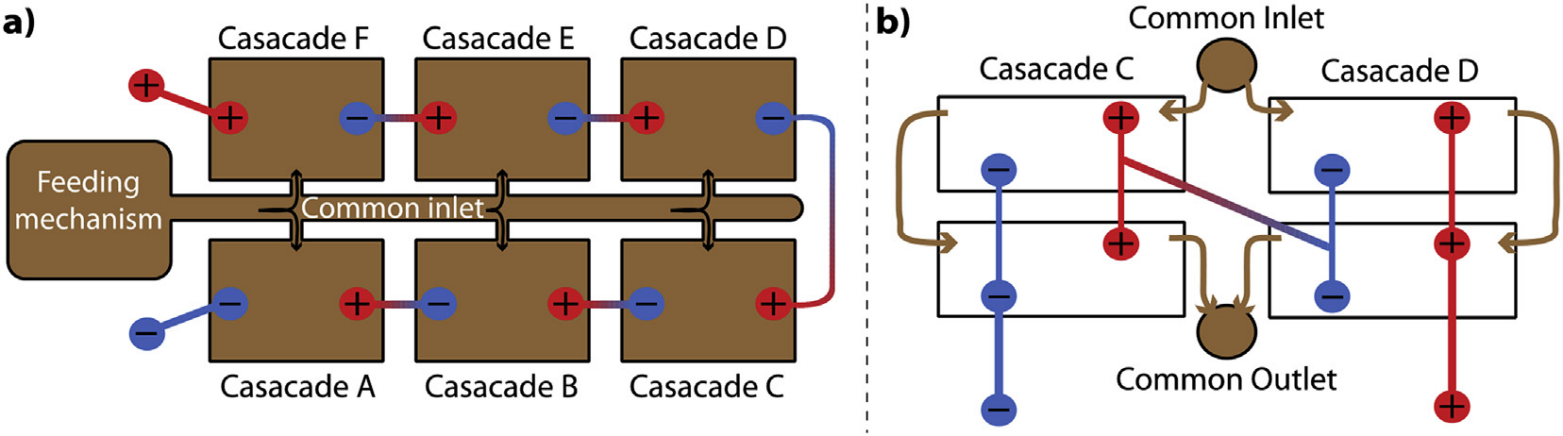
Schematic of the hydraulic and electric connectivity of the MFC stack employed at Glastonbury. (**a**) Top view of the stack illustrating how the 6 cascades were electrically connected in series. (**b**) Side view illustrating how 2 modules within a cascade were electrically connected in parallel and how 2 cascades were connected in series.

To compare the performance with the 2015 trial that used larger modules (33.6L footprint volume; 25L displacement volume) of a different design [13], the same number of SSM-MFC modules was built (n = 12). On site, the height was limited to 45 cm above ground level. Due to this height factor, the need to fit the gravity-feed system (buffer tank, feeding mechanism and MFC stack) and following the results of the laboratory investigation (see §3.1–3.2), the MFC stack comprised 6 cascades of 2 modules each (Fig. 1a). The 2 modules within each cascade were electrically connected in parallel, and the 6 cascades were electrically connected in series (Fig. 1a).

The field trial ran at the Glastonbury 2016 Music Festival (England) from 12/06/2016 to the 02/07/2016. A specially adapted urinal (Dunster House, UK) was installed in the “Sacred Space” (aka “Stone Circle”) field. The 2015 urinal had a theoretical capacity of 9 individuals at any given time, whilst the one described here had a theoretical capacity of 18 people. The urinal comprised 3 troughs collecting the urine from festival-goers, which was then piped towards the MFC system. Next to the urinal, an educational information point was used to engage with the public and explain the ideas and technology behind the project. The collected urine was piped into a buffer tank continuously feeding a passive feeding-mechanism that automatically triggered feed-pulses when 9.8L ± 0.3L of urine was reached within it. Any overflow arriving into the buffer tank was redirected into the nearby soakaway. These 9.8L pulses were channelled into an inlet pipe common to the 6 cascades (Fig. 1a), which then received approximatively 1.6L of neat urine per feed-pulse. The hydraulic retention time (HRT) of the whole set-up was consequently depending on usage and corresponded to 6 feeding pulses.

### 2.2 Energy management circuitry – harvesting and lighting

The purpose of the electronics system used in the Glastonbury 2016 field trial setup was to harvest the energy from the MFC stack to run the urinal's internal lighting so that it could be used outside daylight hours.

**Fig. 2:**
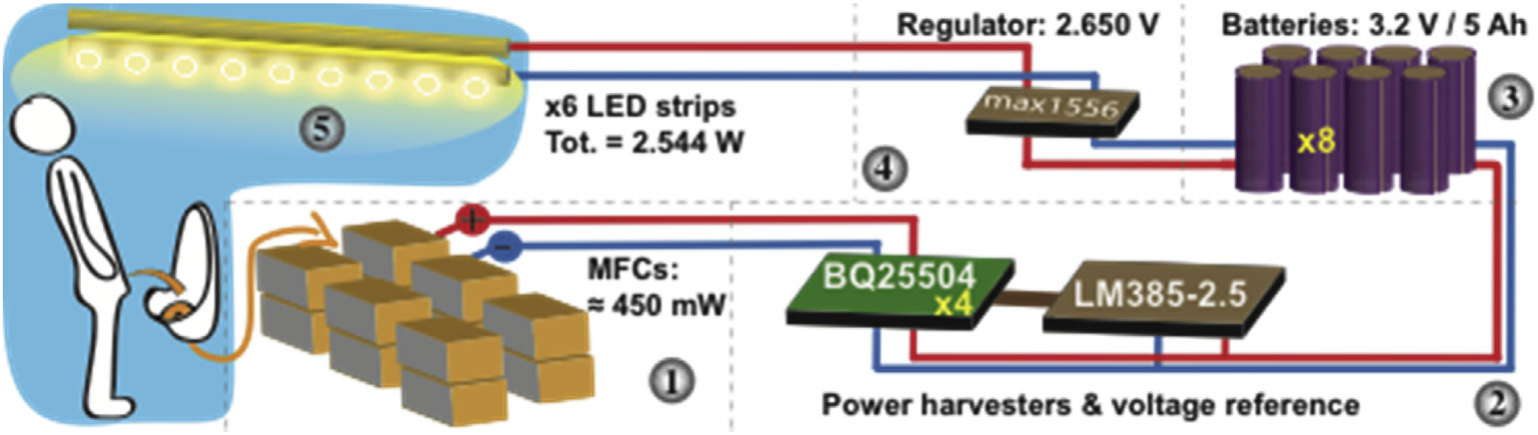
Diagrammatic representation of the MFC system: the urinal feed the MFC stack (stage 1), from which the energy is harvested (stage 2), at a constant voltage by 4 power harvesters connected in parallel, and stored in 8 parallel Lithium Iron Phosphate cylindrical cells (stage 3), connected to a voltage regulator (stage 4) connected to 6 LED strips lighting the urinal ≈9h30 per day (stage 5).

The power harvesters (Fig. 2, stage 2) were boosting MFC voltage to a higher voltage that conform to the battery technology being used for energy storage (Stage 3). This voltage would also have to exceed the voltage required to run the LED lighting system (Fig. 2, stage 5). The BQ25504 energy harvester (Texas Instrument) was used as it has been well tested in previous experiments and provides “off the shelf” functionality. Due to the maximum input power requirement (800 mW) and since a single BQ25504 module has an upper recommended input power of 300 mW, four such systems were connected in parallel. The inclusion of extra harvesters in parallel resulted in benefits of redundancy (should one or more fail) and increased efficiency through the reduction of heat loss through conductors (i.e. copper/winding losses). The following alterations were made to the standard setup of the BQ25504:

• Output voltage settings changed through the replacement of resistors (as described on the BQ25504 datasheet) to conform to the correct voltage levels for the lithium iron phosphate batteries. This effectively alters the over and under voltage limits.
• Setting the harvesters to use an external voltage reference to regulate the MFC input voltage to the desired level (2.70 V). This process involves altering the locations of jumpers (please see BQ25504 datasheet) and including an external voltage reference. The external voltage reference of 2.7 V used for this was an LM385 adjustable micropower voltage reference chip (National Semiconductor).
• Addition of low equivalent-series-resistance 6800 mF capacitors across the battery (BAT) terminals. This increases the efficiency of the harvesting due to reduction in voltage fluctuations and provides a slightly bigger buffer than the one provided by “on board” capacitors.

The battery stack storing the energy (Fig. 2, stage 3) was made up of 8 parallel 3.2 V 5Ah lithium iron phosphate cylindrical cells. Batteries were chosen over capacitors mainly due to cost and energy density. The overall capacity of the batteries was chosen to allow sufficient buffer in case of fluctuations in MFC performance and lighting demand. Lithium iron phosphate was chosen over alternative battery technologies for its balance of safety, cost and energy density.

The fourth stage is the regulator system. This down-regulates the high voltage from the batteries to the lower voltage required to power the LED lights. In this case, a MAX1556 switching regulator (Maxim Integrated) (Fig. 2, stage 4) was used due to its very high efficiency 95% and its ability to directly connect the batteries to the lights should the input level fall below the desired regulation voltage. This means that the lights can continue to work under a much lower voltage if needed. The regulator was set to output 2.650 V, which was sufficient to run the LED lights at the desired intensity (i.e. to comfortably read text on wall posters).

The final stage is the LED lights (Fig. 2, stage 5; 6 x Auralum B01AAHF45I T8 tube lights). These were chosen over the LED lights used in the previous year's field trials due to superior light output for a given energy input. These lights are manufactured to run from mains (240 V AC). Each LED tube light was modified to run at the desired 2.650 V DC voltage. This involved removing the AC-DC converter and re-soldering all of the individual LEDs in parallel within the tube. At the 2.650 V DC, provided by the regulator, an LED strip required 160 mA and therefore 0.424 W, which resulted in a total consumption of 2.544 W.

### 2.3 Data capture and polarisation experiments

Voltage outputs were monitored against time using an Agilent LXI 34972A data acquisition/switch unit (Farnell, UK). Measurements were recorded every 3 min. The polarisation scan was performed on mature modules (i.e. modules had reach electrical steady-state) with the electrical connections (iii) described in §2.1.2. The polarisation sweep was performed manually with a variable resistor (Centrad Boite à Decades de Resistances DR07). Thirteen resistance values were applied and ranged from 500Ω to 2Ω. Each resistor was connected for a period of 30 min in order to limit the influence of the MFC's capacitance [33]. The current *–* in Amperes (A) – was calculated using Ohm's law, *I* = *V*/*R*, where *V* is the measured voltage in Volts (V) and *R* is the known value of the resistor. The power output *P* in Watts (W) was calculated as *P* = *I* x *V.*

During the field trial, a similar setup was monitoring the performances of the stack. A laptop PC and a data logger (LXI 34972A, Keysight Technologies) were recording data and powered by solar panels before the start of the Festival, after which it was powered by the site's local diesel generator. However, as illustrated by the results, the generator stopped on occasion, due to the lack of fuel, hence some data points were not recorded. As the triggering/feeding rate was depending on the urinal usage, a sensor was added in the feeding mechanisms to monitor the discharge pulses.

### 2.4 Chemical analysis

Each sample was collected in duplicate, and analysed in triplicate. Each triplicate was treated and analysed as a separate sample. The chemical oxygen demand (COD) analyses were performed using the potassium dichromate oxidation method (COD HR test vials, Camlab, UK) with 0.2 mL of inlet and outlet samples taken before and after the MFC treatment. The vials were heated at 150 °C during 2 h and cooled to room temperature before the measurements were taken using an MD 200 photometer (Lovibond, UK). Total nitrogen (TN) was measured using Vario Tube Test (0.5–25 mg L^−1^) on diluted samples (1/100) and an MD 500 colorimeter (Lovibond, UK). The ammonium concentration was determined by colorimetric analyses on diluted samples (1/10,000) using tablet reagents (Lovibond, UK) and an MD 500 colorimeter (Lovibond, UK). All the samples were filtered prior to these analyses (0.2 μm pore size membranes). The results are presented as average values, comprising duplicates of each sampling time together with the triplicates of analysis.

## 3 Results and discussion

Coulomb/Coulombic efficiency (CE) and energy recovery are calculated against and normalized to the amount of removed COD (Supplementary information §1.2).

### 3.1 Scaling-up the module size

The modules employed here were twice the size in length and width as the ones tested in earlier laboratory experiments [8,9]. Once stabilised (i.e. reaching steady-state), the power output of a single module reached an absolute value of ≈55 mW at 525 mV and 105 mA. In order to compare its performance with earlier results of the same design, the total displacement volume (Table 1) was used for normalising power density. The volumetric power density of this larger module was of ≈11.5 W m^−3^, which is similar to the ones of a smaller size (i.e. ≈12 W m^−3^)[8]. These preliminary results further confirmed that a SSM-MFC can be scaled-up with limited power density losses. With regard to the total volumetric footprint of the modules, the smaller module had a power density of ≈2.9 W m^−3^ [8,9] whilst the larger one produced ≈2.8 W m^−3^.

**Table 1:** Summary of the performance of “out-of-the-lab” and pilot scale MFCs treating real waste streams. Some data (a) were extracted with permission from Ref. [19]. NER: normalised energy recovery.

Waste stream	Influent COD (mg/L)	Anode Volume^a^ (L)	HRT (h)	COD removal (%)	Power density (W/m^3^)	Coulombic efficiency (%)	Max. NER (kWh/m^3^)	Max. NER (kWh/kgCOD)	References
Domestic Wastewater^b^	279 ± 144	4	11	65-70 max.	1.14	10.7 max.	0.0127	0.0649	[30]
Domestic Wastewater^b^	156 ± 42	96	18	78.8 max.	1.35	1.4 max.	0.0243	0.1976	[31]
Brewery wastewater^b^	3196 ± 978	18.8	156.6	94.5 max.	0.44	13.9 max.	0.0691	0.0229	[19]
Brewery wastewater^b^	3321	90	144	87.6 max.	1	19.1 max.	0.1440	0.0495	[27]
Neat urine*	5586 ± 139	19.2*	44	88*	9.9	3.81	0.3460	0.0704	This Study
Neat urine**	6770 ± 98	57.6	11.7	48	7.31	1.63	0.0860	0.0247	This Study
Neat urine***	–	300***	22	25	1	N/A	0.0220	N/A	[13]

*Laboratory experiment with the cascade of 4 modules. Energy calculated from the area under curve (Fig. 4a, current curve under 2Ω, last 44 h).

**Data from the present field trial at the time of the COD sampling (not the max. power produced). Energy calculated from the area under curve (Fig. 5, current curve 11.7 h prior COD sampling).

***Data extracted from the 2015 field trial [13].

a Volumetric power densities were calculated using anode liquid volume for past studies (b,***) and total electrolyte volume for the present study (*, **).

b The data extracted from Lu et al. 2017 [19] use the max. power densities and the max. Coulombic efficiencies.

### 3.2 Electrical performance of the module when assembled in stacks

**Fig. 3:**
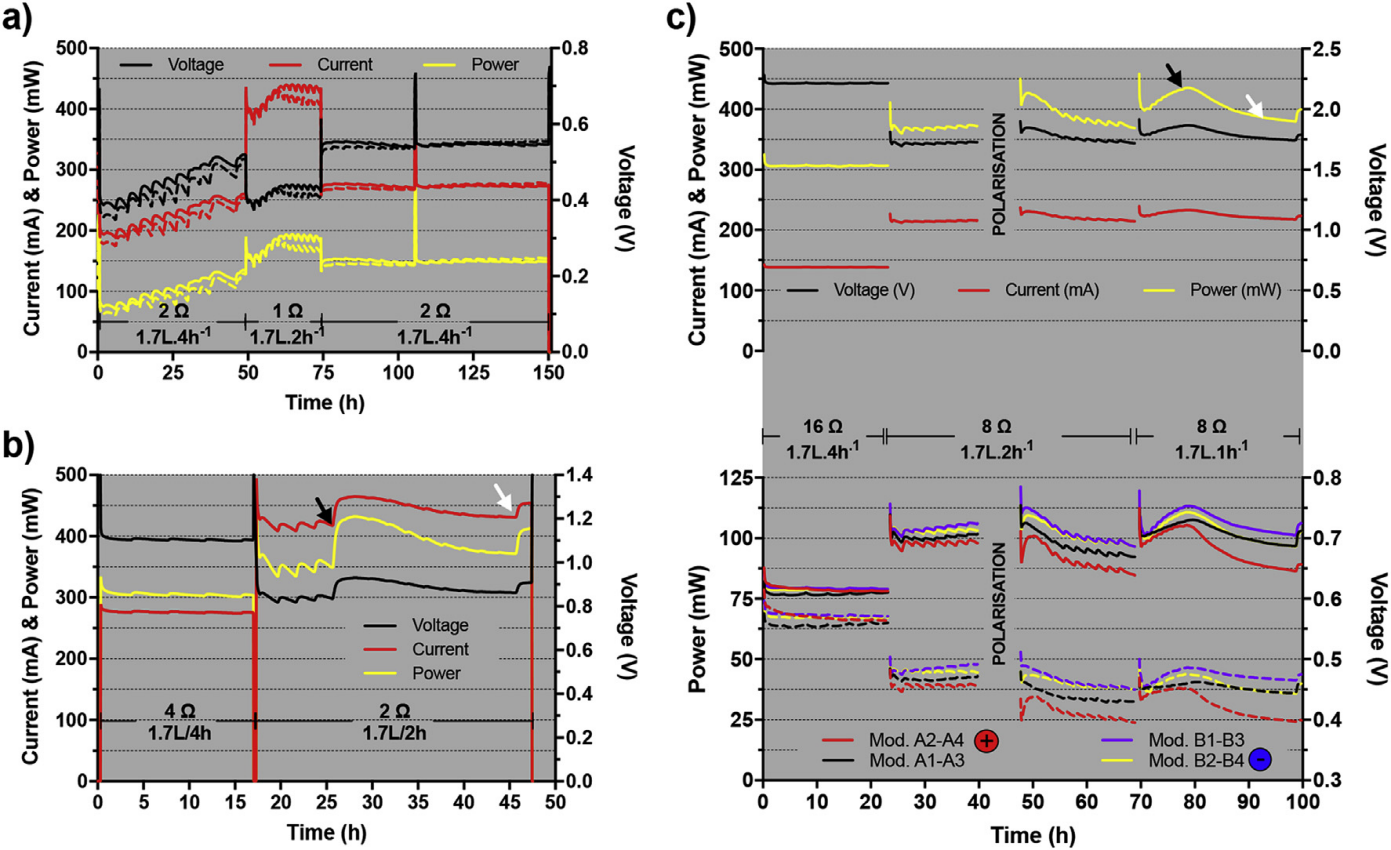
(**a**) Performance of the two electrically independent cascades (i.e. duplicate) under different feeding regimes and corresponding loads: all the modules within each cascade are connected in parallel. (**b**) Electrical behaviour when both cascades are electrically connected in series (1 stack) whilst all the modules within each cascade are connected in parallel. The black arrow indicates a fault on the timer controlling the pump that malfunctioned and pumped the 20 L of fuel in one go. The next feeding occurred 6 h later. The white arrow indicates a manual feed of 10L. (**c**) Electrical performance of the 8 modules stack: modules electrically connected in parallel by pairs, within a single cascade, and the 4 pairs connected in series. The top graph shows the stack behaviour (voltage, current, power) whilst the bottom one shows the modules' behaviour (voltage as dashed lines, power as plain lines). The black arrow indicates when the last feed occurred and the white one indicate a single feed of 5L per cascade.

Because the adjustment of the feeding mechanism was difficult, the pulsated bursts were delivering ≈1.7 L of urine instead of 1.25 L, which is what was used for the single module lab testing. Moreover, due to the availability fuel supply, the tests with similar HRT time were carried out over shorter periods. Compared to the performance of a single module, under the same feeding regime (1.7 L.2 h^−1^), a single cascade of 4 modules electrically connected in parallel was producing, at steady state (from 63 h to 74 h; Fig. 3a), an average of 181 ± 9 mW at 425 ± 11 mV and 425 ± 11 mA (n = 2; Fig. 3a). The power density of a cascade was 18% lower than that of a single module; 2.2 W m^−3^ and 2.7 W m^−3^ footprint volume respectively.

Once the two cascades were connected in series to form a single stack (with all MFC in a cascade connected in parallel), and under a similar feeding regime (1.7 L.2 h^−1^), the average power was of 352 ± 10 mW at 838 ± 11 mV and 419 ± 6 mA (from 17.5 h to 27 h; Fig. 3b). Results show that this one stack of 2 cascades was producing an energy equivalent to the sum of the energy produced by each single cascade. Connecting the two cascades in series increased the voltage (838 ± 11 mV instead of 425 ± 11 mV) and maintained an equivalent current (419 mA and 425 mA, respectively).

Under lower feeding pulses (1 burst of 1.7L every 4 h), the steady-state power output of a single cascade was 151 ± 2 mW at 549 ± 4 mV and 274 ± 2 mA (n = 2 cascades/stacks; Fig. 3a; last 44 h). Again, when electrically connected in series the voltage was doubled, making the average power 305 ± 3 mW at 1105 ± 5 mV and 276 ± 1 mA (t ≈ first 17 h; Fig. 3b). The impact of the timer failure illustrates the dependency on the feeding regime for power (1.7 L.2 h^−1^, black arrow, Fig. 3b). After being fed 10L per cascade at once (black arrow, Fig. 3b), the stack reached a maximum power of 432 mW at 939 mV and 465 mA. These results indicate that these modules have the potential to produce even more power when placed under a higher feeding regime. Twenty hours after the 10 L feed-burst, a steady-state similar to its previous state was reached i.e. ≈ 372 mW at 862 mV and 432 mA (from 42 h to 46 h; Fig. 3b). To confirm this dependence, a 5L per cascade fuel-burst at the end of the run significantly increased the power output to 412 mW (white arrow).

**Fig. 4:**
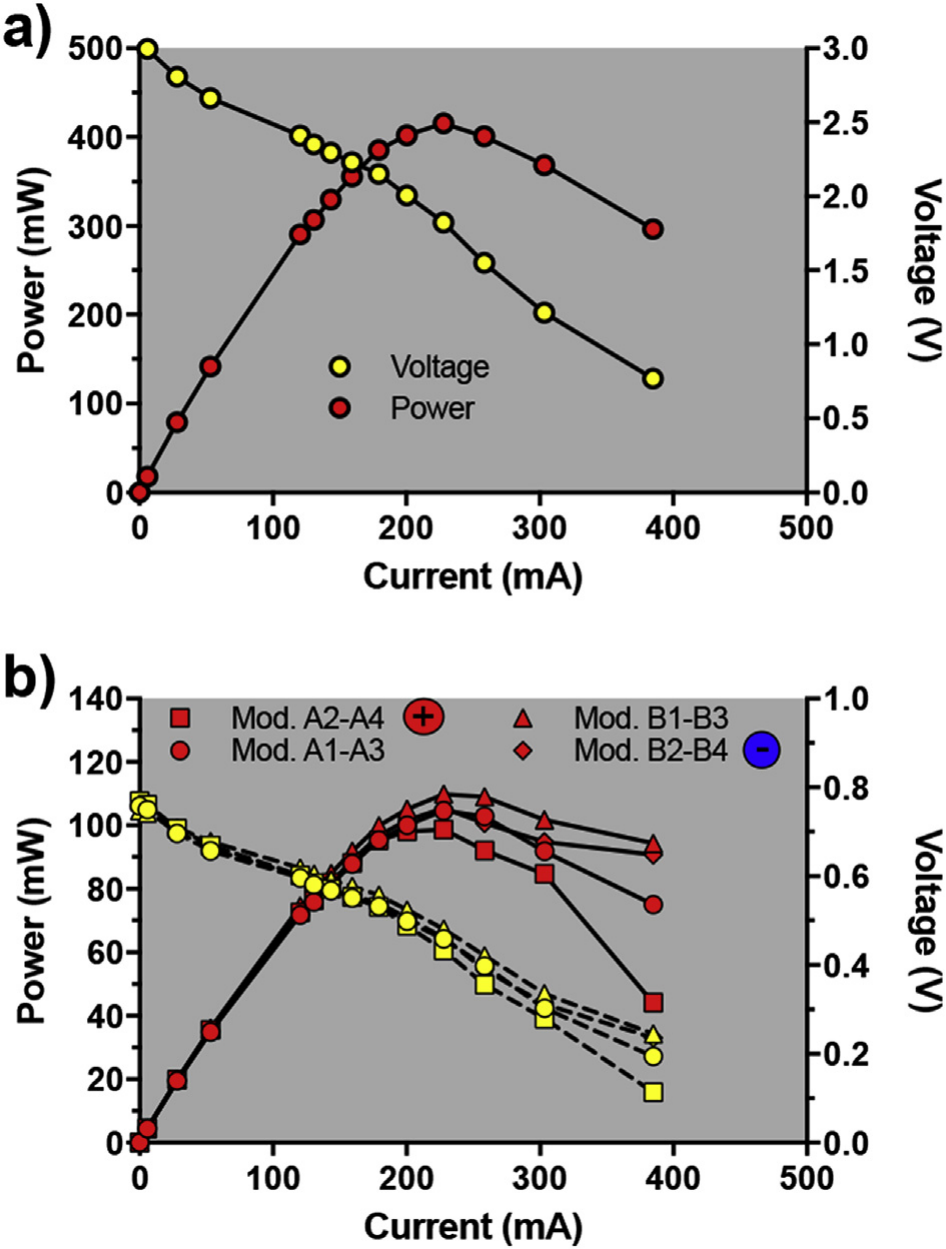
Polarisation sweep results from the stack of 8 modules (**a**), and from the module pairs (**b**).

Following these results, a third electrical configuration was tested. Whilst keeping 2 cascades (i.e. hydraulic configuration), pairs of modules were electrically connected in parallel, and the pairs were then electrically connected in series. However as explained earlier, with fuel being progressively depleted as it travels through the multiple four-module cascades, the downstream modules of the cascade produce less power than the initial ones. To achieve a more balanced system, the first module of a cascade was electrically connected in parallel with the third one, whilst the second was paired with the fourth. The series connection was made by connecting the second pair of the first cascade (A2-A4) with the first one of the same cascade (A1-A3). The latter pair (A1-A3) was then put in series with the first pair of the second cascade (B1-B3), which was then connected in series with the last pair (B2-B4). The positive terminal port of this setup was the pair A2-A4, and the negative port was the pair B2-B4. The hydraulic retention time was then progressively decreased from 1.7L.4 h^−1^ to 1.7L.2 h^−1^ and then to 1.7L.1 h^−1^ (Fig. 3c). The load applied to the stack was adjusted accordingly from 16Ω to 8Ω. The load applied when the stack was under 1.7L.1 h^−1^ was kept to 8Ω since this corresponded to the maximum power transfer point of the stack, as indicated by the polarisation sweep (415 mW, 1824 mV, 228 mA; Fig. 4a).

In addition to the increased power output due to the increased feeding regime, the stack's electrical behaviour matched previous results. With a pulse-fed every 4 h, the stack produced 306 ± 1 mW (from 0 h to 23 h, Fig. 3c), which corresponds to the same power produced when the stack was under the previous configuration shown in Fig. 3b (from 0 h to 17 h). As half the number of modules was electrically connected in parallel, but double the number was connected in series, the current was naturally halved (138 ± 0.1 mA instead of 276 ± 1 mA; Fig. 3c and b) and the voltage doubled (2213 ± 2 mV instead of 1105 ± 5 mV; Fig. 3c and b). Accordingly, under a 2 h pulse-feed regime (HRT ≈ 6 h per module), the stack produced 369 ± 4 mW at 1719 ± 10 mV and 215 ± 1 mA (from 23 h to 40 h, Fig. 3c). When the regime was changed to 1 feeding per hour, the stack ran out of fuel before reaching a steady state (black arrow, Fig. 3c). Nevertheless, during the 2 h following the last feed, the stack produced an average of 434 ± 0.6 mW at 1864 ± 1 mV and 233 ± 0.2 mA (2 h after black arrow, Fig. 3c). Again, these results show that (i) these MFC-modules behave as conventional power sources, and (ii) such a setup would be able to handle a large quantity of urine in proportion to its size.

### 3.3 Glastonbury Music Festival trial: a reliable and effective system

**Fig. 5:**
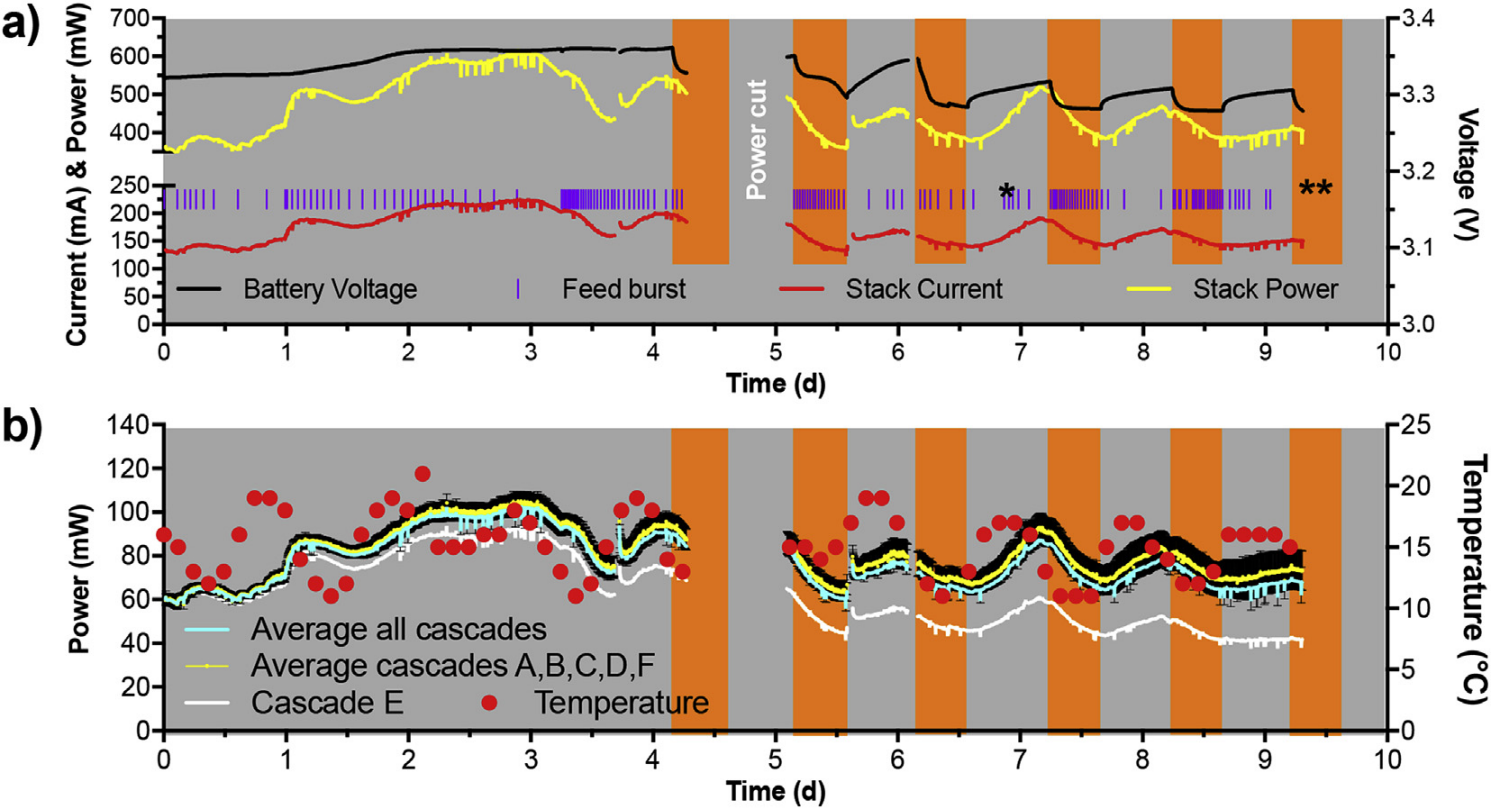
Electrical behaviour of the system before and during the festival. (**a**) Power and current output levels of the stack of 12 modules, voltage of the batteries and feeding-pulses that fed the 6 MFC cascades. (**b**) Average of the absolute power output for all six cascades (light blue); the average power output of the five cascades showing comparable power outputs (A,B,C,D,F; yellow) – error bars show the standard deviation (n = 5 per time point) – the absolute power output of module E showing its drift from the average (white); and the air temperature (red points). Orange bars indicate the periods of illumination of the urinal (from ≈ 9pm to ≈ 7am). * indicates when samples were taken for the COD and nitrogen analysis. (For interpretation of the references to color in this figure legend, the reader is referred to the Web version of this article.)

During the first 3 days, the stack installed at the Glastonbury Music festival behaved as expected from the results obtained during the laboratory tests. The power output gradually increased (2 days; Fig. 5a) to a steady state of 590 ± 12 mW at 217 ± 5 mA (Fig. 5a), which held for 28 h until most of the festival attendees had arrived (from day 3 to day 5). This corresponds to 50 KJ of electrical energy or 14 Wh for every day that the system was running. After adapting to the new feeding regime and conditions (i.e. passive system), a new steady-state was reached from days 5–9 (Fig. 5a). During this period, which includes all variations, the average power generated from the stack was of 424 ± 36 mW at 156 ± 12 mA (Fig. 5a).

**Fig. 6:**
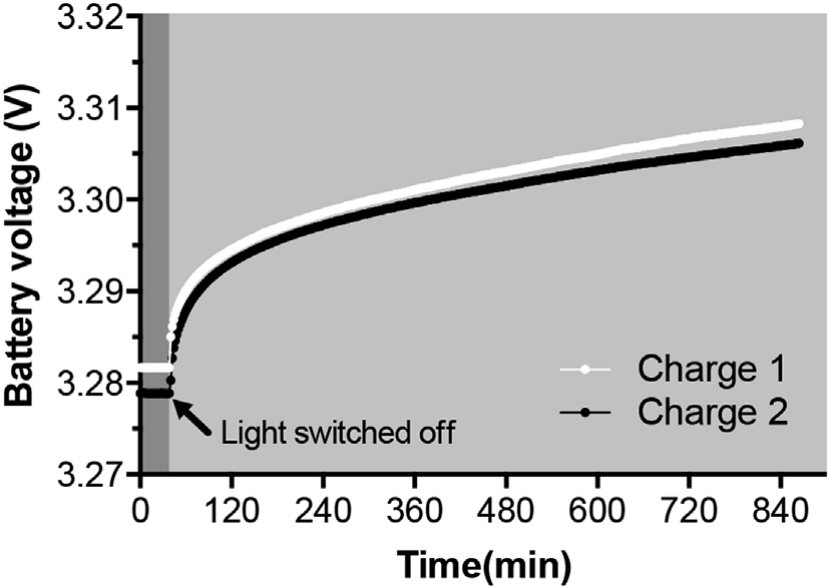
Battery bank voltage during the charging phase. Charge 1 was during day 8 and charge 2 during day 9 (1 point every 2 min). Dark grey area indicates the period when the lights were on, prior being switched off (light grey area).

The majority of the festival attendees arrived on the third day. It is likely that both temperature and feeding patterns would have contributed to the diurnal variations. During the night from day 7 to day 8, the feeding rate was relatively high (≈1 feed every 25 min; Fig. 5a), and the consequent power level variation more pronounced than during the previous night. Since the temperature was higher during day 6 than day 7 (Fig. 6b), the difference in power fluctuation between these two successive days could thus be attributed to the feeding regimes. Results therefore indicate that for this system with this specific module size, a feeding rate of 1 feed every 25 min was too high (decrease of power; Fig. 5a), and a feeding rate of 1 feed every 90 min seems optimum (stable and/or increasing power production; Fig. 5a). These feeding rates correspond, for the whole system (57.6L), to a hydraulic retention time of ≈2h30 min (≈559L.d^−1^) and ≈9 h (≈155L.d^−1^), respectively. It is important to note that this is a field test with a passive feeding system (i.e. no energy consumption). Hence, the feeding regimes depend on the uncontrolled usage. The observed decrease of power due to frequent feed-pulses (1 feed every 25 min) could be of two origin. Either the feeding rate, which mix the electrolyte and disrupts the electrochemical stratification, is faster than the capacity of the system to re-stratify (system's resilience) – and/or the feeding rate is higher than the microbial population's growth rate (washing out phenomenon) [34]. These results together with the laboratory tests lead to the conclusion that the optimum HRT was comprised between 2 h30min, which is too short, and ≈9 h, which might be too long.

With regard to the total volumetric footprint of the MFC stack, the power density was 1.7 W m^−3^. The results of the 2015 trial only reported the footprint volume of each module (i.e. total of 403.2 L), without taking into account the air-gaps between modules [13]. Hence, in order to make a direct comparison for this year, the air gap volume (6 L) was also discounted from the total footprint volume. Under these normalising conditions, the power density obtained is 2.45 W m^−3^. In comparison to the 2015 setup (mean of 300 mW corresponding to 0.74 W m^−3^), the power density of the 2016 setup was improved by 331%. When normalising by the displacement volume (≈58 L and 300L, respectively), results show a 731% improvement from the SSM-MFC design (Table 1).

The calculation of the Coulombic efficiency was done with whole volume of electrolyte was taken into account. When considering a stack of MFCs, the equation has to be modified to reflect the electrical connections. Thus, for a stack with a series configuration, the current – in equation (1) (Supplementary Information)– should be multiplied by the number of MFCs in series. Considering these parameters, the coulombic efficiency obtained are 3.81%, 3.43% and 1.63% for a HRT of 44 h, 22 h and 11.7 h, respectively. However, NER values are more suited to go beyond coulombic efficiency and better reflect energy production by emphasising the fact that MFC are wastewater treatment systems [35,36]. Using equations (2) and (3) the obtained NER values reflecting the treatment capacity of the system for the HRT of 44 h, 22 h and 11.7 h, are 0.346 ± 0.005, 0.207 ± 0.010 and 0.086 ± 0.007 kWh.m^−3^, respectively. With regard to the conversion efficiency of the system, the NER values for the HRT of 44 h, 22 h and 11.7 h, are 0.070 ± 0.001, 0.049 ± 0.002 and 0.025 ± 0.002 kWh.kg-COD^−1^, respectively. Since the system presented here was fuelled by neat urine, comparison with previous systems fuelled with domestic or brewery wastewaters is challenging. Nonetheless, the system presented here shows similar performance to what has been published (Table 1). Moreover, even though the power densities were the highest amongst the compared ones (Table 1), the NER values were in the same range, which strengthen the notion that NER values would be more appropriate to compare MFC system in the context of practical implementation [36].

At the beginning of the field trial all the cascades (A, B, C, D, E, F; Fig. 1) had comparable power outputs. However, the performance of cascade E, which was already the weakest of the system, started diverging from the other 5 cascades beginning of day 4. The power outputs levels of these cascades were always comparable throughout the trial (±5.1 mW on day 5 and ± 9 mW on day 9, Fig. 5b). However, the drift from cascade E decreased the power output of the stack by 8% (difference between average of five and average of six modules). It should be noted that it is difficult to evaluate the real impact of this drift, since it could have inhibited the activity of the other connected modules. Hence, the power output of the stack could have been higher than 8%. In comparison with the significant power decrease of module E (43% less power than the average on day 9), the 8% power decrease illustrates that the MFC stack was a robust power source, even with an “underperforming” cascade.

The energy was stored in a battery bank during day time and powered the lights at night. The duty cycle was of ≈14h30 charge time and ≈9h30 discharge time. However, as can be seen in Fig. 6, the voltage level of the battery was decreasing. This result indicates that during a 24 h period the lighting system was consuming more energy than that produced. Assuming a hypothetical 100% efficiency from the energy management system (i.e. harvester, battery and output voltage regulator) the 6 LED strips were consuming ≈87 kJ of electrical energy (2.544 W during 9h30) whilst the MFC stack was producing between ≈52 kJ (≈0.600 W continuously; days 0–4) and ≈37 kJ (≈0.425 W continuously; days 6–9). The reason for the decrease in power is the irregular feeding during the festival, as already discussed. To balance the system and achieve self-sustainability, whilst delivering the same service (2.544 W lighting during 9.5 h), since the MFC stack could process 220L of urine out of the 1,200L received daily from the urinal, the size of the stack could have been doubled to produce more power than that consumed by the LEDs.

### 3.4 Treatment capacity of SSM-MFCs

**Fig. 7:**
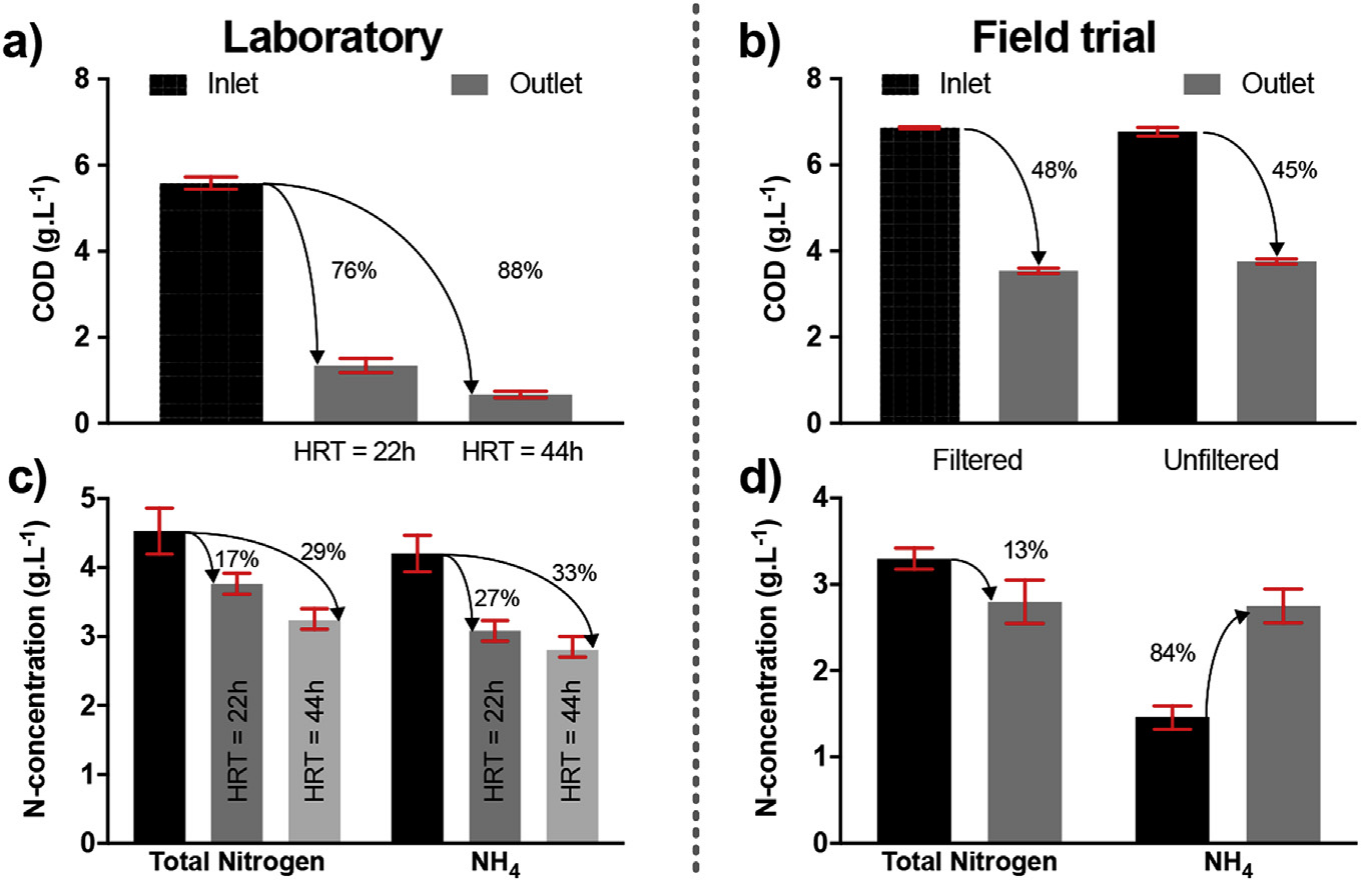
Chemical Oxygen Demand (COD) (**a, b**), total nitrogen and ammonium reduction (**c, d**) in treated urine stream during laboratory investigation (**a, c**) and during the field trial at 11h40 HRT (**b, d**). The HRTs in (**a**) were obtained by changing the pulse-feed duty cycles: 22 h and 44 h correspond to 1.7L.2 h^−1^ and 1.7L.4 h^−1^, respectively (Fig. 3).

During the laboratory investigation, the HRT applied to the MFC cascades was longer than the one adjusted for the PEE POWER^®^ system during the festival. This was due to the challenges of collecting sufficient fuel in the lab, which is why for the lab trials, the systems had a 22 h and 44 h HRT applied. As explained earlier, feeding regime and produced power are correlated. This is why the applied loads differed for the different HRTs (Fig. 3). Under these conditions, if longer HRT displayed higher nutrient removal, the correlation was not linear (Fig. 7a). During the Glastonbury trial, the COD decreased by 48% (Fig. 7b)– with an HRT of 11h40 min at the time of analysis – and the total nitrogen (TN) content had decreased by 13% (Fig. 7a). Compared to the previous trial (2015) [37], the COD removal for the current trial was 92% higher, with half the HRT.

**Fig. 8:**
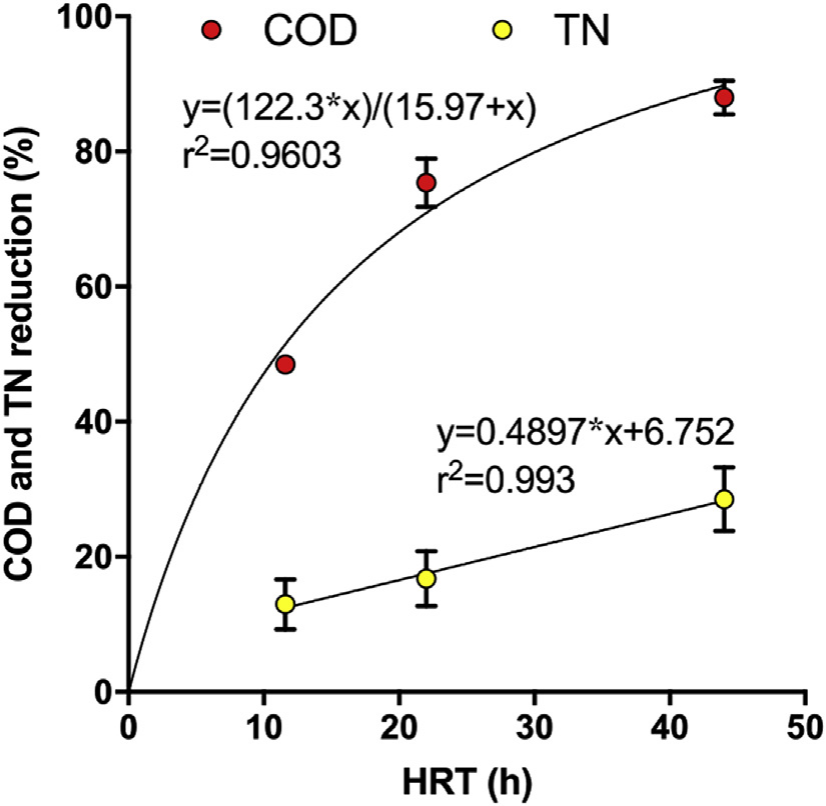
COD and total nitrogen (TN) removal percentage depending on the hydraulic retention time of a cascade. The first data points (HRT = 11h40) correspond to the samples taken from the Glastonbury trial, whilst the second (HRT = 22 h) and the third (HRT = 44 h) correspond to the laboratory testing (i.e. data merged from two different experiments).

The nitrogen removal was ≈50% lower than the previous trial, but this was recorded for an HRT which was 50% shorter; it can therefore be assumed that the nitrogen removal rates were similar, and in both cases, were slower than the corresponding COD removal rates. The treatment efficiency of the stack comprising 2 cascades of 4 modules (laboratory experiment) was plotted against the HRT, together with the results of the Glastonbury trial (Fig. 8). This projection indicates that the higher the HRT the more efficient the waste treatment. Longer HRT could be achieved either by increasing the number of modules in a cascade or by adjusting the pulse-feeding regime.

Compared to the industry standards (92% COD and 20% TN reduction) [38], the results from the 44 h HRT are relatively close (88% COD and 29% TN reduction). However, in our case we were treating a much more concentrated waste stream (5500–6800 mg COD.L^−1^) compared to the mixed wastewater streams received by a municipal wastewater treatment plant (e.g. rain water, grey water, black water). Regarding the legal maximum discharge concentration in the European Union (conc. <125 mg COD·L^−1^; <10–20 mg TN L^−1^) [39], the 4 module stack with an HRT of 44 h does not meet the requirements (≈670 mg COD·L^−1^; ≈3230 mg TN L^−1^). To reach the legal acceptable limits, the COD should be decreased by 98%. According to the Michaelis-Menten equation that fits the COD removal projection (Fig. 8), it can be hypothesised that the required COD discharge concentration could be reached with a HRT of ≈64 h. Since the TN removal rate is not yet substrate-limited (linear correlation, Fig. 8), it is not possible to give an estimation of the optimal HRT.

## 4 Conclusions

The results from the Glastonbury trial demonstrate that the performance of the system followed the projections stemming from the laboratory investigation. With regard to the amount of urine produced daily (∼1200L), only ∼220L fuelled the MFCs stack whilst the remaining ∼980L was unused and piped away through the system's overflow. To accommodate such an amount, the MFC stack should have comprised 60–70 modules. Since the power produced by 12 modules (424 ± 36 mW) was below the requirement for the 2.544 W lighting system, it can be projected that a stack of 60–70 modules would power a lighting system of ≈6–7 W. In such a case, the challenge would be to homogenise fuel distribution. For a PEE POWER^®^ system which would directly discharge the effluent to the environment, the results indicate that: i) the legal COD levels could be reached with an HRT of ∼64 h, and ii) a 4-modules cascades is yet insufficient to reach the total nitrogen concentrations allowing such direct discharge. However, to the authors' best knowledge, the MFC is the only biotechnology able to directly treat neat urine – with no dilution at any step of the process, and without inhibition due to high nitrogen concentrations – within this level of efficiency and with energy production (i.e. not energy consumption), which is the case for the majority of other biological technologies or processes. Overall, although results from this study show that there is still room for improvement before any commercial deployment, the MFC technology is sufficiently mature to be introduced as a carbon neutral pre-treatment system that would positively impact liquid waste stream management in urban, as well as rural areas. Moreover, the results in this study strengthen the thesis that MFCs can act as a power supply in decentralised areas.
