# Supplementary material for: PEE POWER® urinal II – Urinal scale-up with microbial fuel cell scale-down for improved lighting
Source: J Power Sources. 2018 Jul 15;392:150–8. doi: 10.1016/j.jpowsour.2018.02.047 (PMC5989813; doi:10.1016/j.jpowsour.2018.02.047)
Supplement: Online data [file mmc1.docx]

**Supplementary information for: *Pee Power^®^ urinal II – urinal scale-up with microbial fuel cell scale-down for improved lighting***

Xavier Alexis Walter^1^*, Irene Merino-Jimenez^1^, John Greenman^1^ and Ioannis Ieropoulos^1^*

^1^ Bristol BioEnergy Centre, Bristol Robotics Laboratory, T-Block, Frenchay Campus, University of the West of England (UWE), Bristol, BS16 1QY, United Kingdom. Fax: +44(0)1173283960;

**Material and methods**

*1.1 Glastonbury 2016 system configuration*

The size of individual modules had the following external dimensions: 400 mm length, 300 mm width and 170 mm height (“large module”). A total of 38 MFCs were inserted within this volume and all were electrically connected in parallel. The total footprint volume of a module was 20.4L, of which 11.2L was occupied by the MFCs (i.e. internal volume). The rest of the volume was occupied by air since the upper 5cm of each box served as a support for upstream modules, resulting in a displacement volume of 4.8L of electrolyte (Fig. 1a).

On site, the height was limited to 45 cm above ground level. Due to this height factor, the need to fit the gravity-feed system (buffering tank, feeding mechanism and MFC stack) and the MFC stack comprised 6 cascades of 2 modules each. The 2 modules within each cascade were electrically connected in parallel, and the 6 cascades were electrically connected in series.


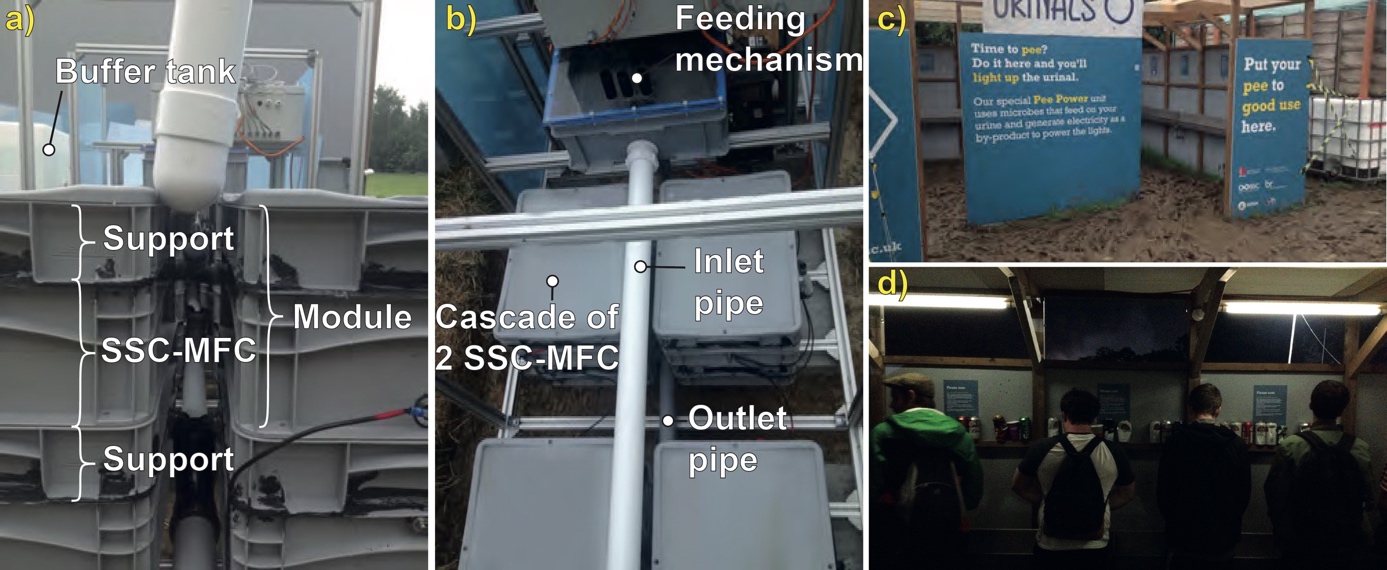


**Figure 1:** Pictures illustrating the MFC installation during the field trial. (**a**) side view of the cascade. (**b**) Top view of four of the cascades and the feeding mechanism. (**c**) Early morning view of the urinal and (**d**) early evening at the urinal with the lights turned on (≈ 11 pm).

*1.2. Power normalisation*

The coulombic efficiency (CE) was calculated using the half reaction taking place in the cathode – i.e. oxygen reduction reaction (ORR) when using an air-breathing cathode [1]:

$$C_{E}=\frac{M\cdot I}{F\cdot e\cdot q\cdot\Delta COD} (1)$$

Where *M* is the molecular weight of the substrate (*O_2_*, 32 g.mol^-1^), *I* (A) is the current, *F* (96485 C.mol^-1^) is the Faraday’s constant, *e* (4) is the number of electrons transferred during the ORR, *q* is the flow rate (L.s^-1^) and $\Delta COD$ (g.L^-1^) is the COD difference between influent and effluent.

To better reflect the performance of the system as a whole, the normalised energy recovery (NER), as defined by Ge *et al.* [2], was also calculated. The NER is expressed under two units. The power is normalised either by the flow rate to the energy produced per volume treated (kWh.m^-3^), or by the flow rate and the difference COD concentration to reflect the energy produced per amount of COD degraded (kWh.kg-COD^-1^). The NER values are calculated as follows for systems under continuous feeding conditions [2]:

$$NER=P/q (2)$$

$$NER=P/\left( q.\Delta COD \right) (3)$$

Where *P* is the power (W), *q* is the flow rate (m^3^.h^-1^) and $\Delta COD$ (kg.m^-3^) is the COD difference between influent and effluent.

[1] Logan BE, Hamelers B, Rozendal RA, Schrorder U, Keller J, Freguia S, Aelterman P, Verstraete W and Rabaey K. Microbial fuel cells: Methodology and technology. Environ. Sci. Technol. 2006;40:5181-5192

[2] Ge Z, Li J, Xiao L, Tong YR and He Z. Recovery of Electrical Energy in Microbial Fuel Cells. Environ. Sci. Technol. Lett. 2014;1:137-141
